# 1-[(4-Bromo­phen­yl)(morpholin-4-yl)meth­yl]naphthalen-2-ol

**DOI:** 10.1107/S1600536812002620

**Published:** 2012-01-31

**Authors:** Qun Zhao

**Affiliations:** a School of Pharmaceutical Sciences, Nanjing University of Chinese Medicine, Nanjing 210046, People’s Republic of China

## Abstract

The title compound, C_21_H_20_BrNO_2_, was obtained from a condensation reaction of 4-bromo­benzaldehyde, 2-naphthol and morpholine. The mol­ecular conformation is stabilized by an intra­molecular O—H⋯N hydrogen bond, closing a six-membered ring. The dihedral angle between the naphthalene ring system and the benzene ring is 76.72 (8)°. In addition to the intra­molecular hydrogen bond, the O—H groups of centrosymmetrically related mol­ecules form short inter­molecular H⋯O contacts of 2.59 Å. These mol­ecules are also linked by pairs of C—H⋯O inter­actions, generating an *R*_2_^2^(14) motif.

## Related literature

For applications of Betti-type reactions, see: Lu *et al.* (2002 ▶); Xu *et al.* (2004 ▶); Wang *et al.* (2005 ▶).
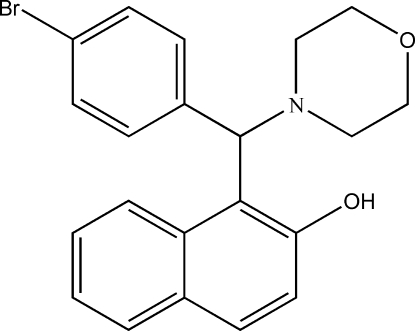


## Experimental

### 

#### Crystal data

C_21_H_20_BrNO_2_
*M* *_r_* = 398.29
Monoclinic, 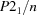

*a* = 11.024 (2) Å
*b* = 12.119 (2) Å
*c* = 13.875 (3) Å
β = 104.55 (3)°
*V* = 1794.2 (6) Å^3^
*Z* = 4
Mo *K*α radiation
μ = 2.31 mm^−1^
*T* = 293 K
0.2 × 0.2 × 0.2 mm

#### Data collection

Rigaku Mercury2 diffractometer
Absorption correction: multi-scan (*CrystalClear*; Rigaku, 2005 ▶) *T*_min_ = 0.802, *T*_max_ = 1.000
18164 measured reflections
4108 independent reflections
3021 reflections with *I* > 2σ(*I*)
*R*_int_ = 0.059

#### Refinement

*R*[*F* ^2^ > 2σ(*F* ^2^)] = 0.045
*wR*(*F* ^2^) = 0.102
*S* = 1.08
4108 reflections
226 parameters
H-atom parameters constrained
Δρ_max_ = 0.24 e Å^−3^
Δρ_min_ = −0.48 e Å^−3^

### 

Data collection: *CrystalClear* (Rigaku, 2005 ▶); cell refinement: *CrystalClear* (Rigaku, 2005 ▶); data reduction: *CrystalClear*; program(s) used to solve structure: *SHELXS97* (Sheldrick, 2008 ▶); program(s) used to refine structure: *SHELXL97* (Sheldrick, 2008 ▶); molecular graphics: *SHELXTL* (Sheldrick, 2008 ▶); software used to prepare material for publication: *SHELXTL*.

## Supplementary Material

Crystal structure: contains datablock(s) I, global. DOI: 10.1107/S1600536812002620/gk2449sup1.cif

Structure factors: contains datablock(s) I. DOI: 10.1107/S1600536812002620/gk2449Isup2.hkl

Supplementary material file. DOI: 10.1107/S1600536812002620/gk2449Isup3.cml

Additional supplementary materials:  crystallographic information; 3D view; checkCIF report

## Figures and Tables

**Table 1:** Hydrogen-bond geometry (Å, °)

*D*—H⋯*A*	*D*—H	H⋯*A*	*D*⋯*A*	*D*—H⋯*A*
O1—H1*A*⋯N1	0.82	1.93	2.622 (3)	142
C13—H13*A*⋯O1^i^	0.93	2.53	3.357 (4)	148

Symmetry code: (i) 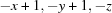.

**Fig. 1.**
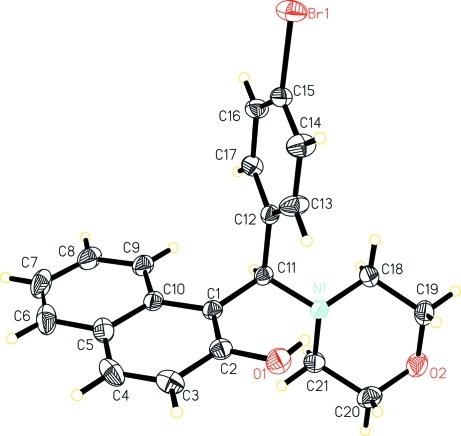
Perspective structure of the title compound, showing the atom-numbering scheme. Displacement ellipsoids are drawn at the 30% probability level.

**Fig. 2.**
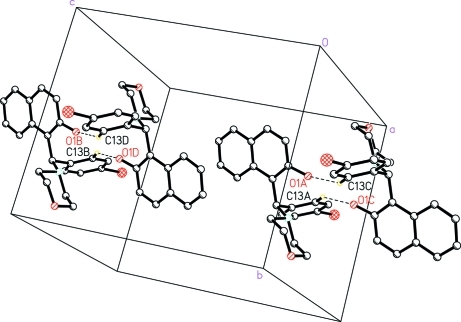
The crystal packing of the title compound viewed along the a axis showing C-H···O hydrogen bonds.

## supplementary crystallographic information

### Comment

Betti-type reaction is an important method to synthesize chiral ligands and by this method many unnatural homochiral amino-phenol compounds have been obtained (Lu *et al.* 2002; Xu *et al.* 2004; Wang *et al.* 2005). Herein we report the synthesis and crystal structure of the title compound, 1-((4-bromophenyl)(morpholino)methyl)naphthalen-2-ol (Fig. 1).

In the title compound (Fig. 1) bond lengths and angles have normal values. The dihedral angle between the naphthylene ring system and the benzene ring is 76.72 (8)°. In the solid state the molecules are linked into centrosymmetric *R*^2^_2_(14) dimers by a simple C–H···O interaction (Fig. 2). In addition to intramolecular hydrogen bond,the O-H groups of centrosymmetrically related molecules form short intermolecular H···O contacts of 2.59 Å. The molecular conformation is stabilized by O–H···N a hydrogen bonding, Table 1.

### Experimental

4-Bromobenzaldehyde (2.76 g, 0.015 mol) and morpholine (1.305 g, 0.015 mol) were added to 2-naphthol (2.16 g, 0.015 mol) without solvent under nitrogen. The temperature was raised to 120°C in one hour gradually and the mixture was stirred at this temperature for 12 h. The system was treated with 30 ml of 95% ethanol and cooled. The precipitate was filtered and washed with a small amount of 95% ethanol. The title compound was isolated using column chromatography (petroleum ether/ethyl acetate 3/1). The melting point of the title compound is 443 K. Single crystals suitable for X-ray diffraction analysis were obtained from slow evaporation of a solution of the title compound in ethyl acetate at room temperature.

### Refinement

H atoms were positioned geometrically and refined using a riding model, with C—H = 0.93–0.97 Å and *U*_iso_(H) = 1.3–1.6*U*_eq_(C).

### Crystal data

**Table:**

C_21_H_20_BrNO_2_	*F*(000) = 816
*M**_r_* = 398.29	*D*_x_ = 1.474 Mg m^−^^3^
Monoclinic, *P*2_1_/*n*	Mo *K*α radiation, λ = 0.71073 Å
Hall symbol: -P 2yn	Cell parameters from 4108 reflections
*a* = 11.024 (2) Å	θ = 2.7–27.5°
*b* = 12.119 (2) Å	µ = 2.31 mm^−^^1^
*c* = 13.875 (3) Å	*T* = 293 K
β = 104.55 (3)°	Prism, colorless
*V* = 1794.2 (6) Å^3^	0.2 × 0.2 × 0.2 mm
*Z* = 4	

### Data collection

**Table:**

Rigaku Mercury2 diffractometer	4108 independent reflections
Radiation source: fine-focus sealed tube	3021 reflections with *I* > 2σ(*I*)
graphite	*R*_int_ = 0.059
Detector resolution: 13.6612 pixels mm^-1^	θ_max_ = 27.5°, θ_min_ = 3.0°
φ scan	*h* = −14→14
Absorption correction: multi-scan (*CrystalClear*; Rigaku, 2005)	*k* = −15→15
*T*_min_ = 0.802, *T*_max_ = 1.000	*l* = −17→18
18164 measured reflections	

### Refinement

**Table:**

Refinement on *F*^2^	Primary atom site location: structure-invariant direct methods
Least-squares matrix: full	Secondary atom site location: difference Fourier map
*R*[*F*^2^ > 2σ(*F*^2^)] = 0.045	Hydrogen site location: inferred from neighbouring sites
*wR*(*F*^2^) = 0.102	H-atom parameters constrained
*S* = 1.08	*w* = 1/[σ^2^(*F*_o_^2^) + (0.0357*P*)^2^ + 0.6882*P*] where *P* = (*F*_o_^2^ + 2*F*_c_^2^)/3
4108 reflections	(Δ/σ)_max_ = 0.001
226 parameters	Δρ_max_ = 0.24 e Å^−^^3^
0 restraints	Δρ_min_ = −0.48 e Å^−^^3^

### Special details

**Table:**

Geometry. All e.s.d.'s (except the e.s.d. in the dihedral angle between two l.s. planes) are estimated using the full covariance matrix. The cell e.s.d.'s are taken into account individually in the estimation of e.s.d.'s in distances, angles and torsion angles; correlations between e.s.d.'s in cell parameters are only used when they are defined by crystal symmetry. An approximate (isotropic) treatment of cell e.s.d.'s is used for estimating e.s.d.'s involving l.s. planes.
Refinement. Refinement of *F*^2^ against ALL reflections. The weighted *R*-factor *wR* and goodness of fit *S* are based on *F*^2^, conventional *R*-factors *R* are based on *F*, with *F* set to zero for negative *F*^2^. The threshold expression of *F*^2^ > σ(*F*^2^) is used only for calculating *R*-factors(gt) *etc*. and is not relevant to the choice of reflections for refinement. *R*-factors based on *F*^2^ are statistically about twice as large as those based on *F*, and *R*- factors based on ALL data will be even larger.

### Fractional atomic coordinates and isotropic or equivalent isotropic displacement parameters (Å^2^)

**Table:**

	*x*	*y*	*z*	*U*_iso_*/*U*_eq_	
Br1	1.13818 (3)	0.46528 (3)	0.17657 (3)	0.05695 (13)	
O1	0.44848 (19)	0.46722 (16)	0.08452 (13)	0.0495 (5)	
H1A	0.4865	0.5249	0.0822	0.074*	
N1	0.54544 (19)	0.65629 (17)	0.15761 (14)	0.0339 (5)	
C15	0.9785 (2)	0.5011 (2)	0.1973 (2)	0.0387 (6)	
C16	0.9716 (2)	0.5484 (2)	0.2853 (2)	0.0430 (7)	
H16A	1.0439	0.5604	0.3356	0.052*	
O2	0.41804 (19)	0.84827 (16)	0.06130 (14)	0.0529 (5)	
C17	0.8560 (2)	0.5779 (2)	0.29851 (19)	0.0383 (6)	
H17A	0.8513	0.6114	0.3578	0.046*	
C5	0.5138 (3)	0.3376 (2)	0.3700 (2)	0.0465 (7)	
C1	0.5505 (2)	0.4867 (2)	0.25927 (18)	0.0343 (6)	
C11	0.6207 (2)	0.5910 (2)	0.24246 (17)	0.0328 (5)	
H11A	0.6352	0.6364	0.3027	0.039*	
C10	0.5719 (2)	0.4394 (2)	0.35761 (19)	0.0373 (6)	
C9	0.6502 (3)	0.4891 (3)	0.44318 (19)	0.0462 (7)	
H9A	0.6889	0.5560	0.4374	0.055*	
C12	0.7474 (2)	0.5589 (2)	0.22564 (17)	0.0320 (5)	
C3	0.4124 (3)	0.3329 (2)	0.1943 (2)	0.0472 (7)	
H3A	0.3587	0.2986	0.1402	0.057*	
C14	0.8724 (3)	0.4807 (3)	0.1230 (2)	0.0508 (8)	
H14A	0.8780	0.4486	0.0634	0.061*	
C21	0.4301 (2)	0.6992 (2)	0.1799 (2)	0.0422 (6)	
H21A	0.4517	0.7497	0.2359	0.051*	
H21B	0.3823	0.6388	0.1977	0.051*	
C18	0.6141 (3)	0.7518 (2)	0.1316 (2)	0.0447 (7)	
H18A	0.6905	0.7268	0.1157	0.054*	
H18B	0.6370	0.8018	0.1878	0.054*	
C6	0.5383 (3)	0.2888 (3)	0.4655 (3)	0.0652 (10)	
H6A	0.5012	0.2216	0.4733	0.078*	
C13	0.7575 (3)	0.5088 (3)	0.1383 (2)	0.0518 (8)	
H13A	0.6852	0.4937	0.0888	0.062*	
C2	0.4726 (2)	0.4315 (2)	0.18050 (19)	0.0379 (6)	
C4	0.4320 (3)	0.2876 (2)	0.2861 (3)	0.0540 (8)	
H4A	0.3910	0.2225	0.2943	0.065*	
C20	0.3525 (3)	0.7583 (3)	0.0899 (2)	0.0537 (8)	
H20A	0.3291	0.7067	0.0349	0.064*	
H20B	0.2761	0.7853	0.1044	0.064*	
C19	0.5324 (3)	0.8112 (3)	0.0430 (2)	0.0530 (8)	
H19A	0.5778	0.8740	0.0265	0.064*	
H19B	0.5140	0.7619	−0.0139	0.064*	
C8	0.6695 (3)	0.4395 (3)	0.5347 (2)	0.0603 (9)	
H8A	0.7202	0.4741	0.5902	0.072*	
C7	0.6148 (4)	0.3382 (3)	0.5461 (3)	0.0701 (11)	
H7A	0.6306	0.3047	0.6083	0.084*	

### Atomic displacement parameters (Å^2^)

**Table:**

	*U*^11^	*U*^22^	*U*^33^	*U*^12^	*U*^13^	*U*^23^
Br1	0.03958 (18)	0.0596 (2)	0.0768 (3)	0.00364 (14)	0.02412 (15)	−0.00697 (16)
O1	0.0537 (12)	0.0564 (12)	0.0335 (10)	−0.0090 (10)	0.0017 (9)	−0.0046 (9)
N1	0.0327 (11)	0.0389 (12)	0.0301 (11)	0.0051 (9)	0.0080 (9)	0.0044 (9)
C15	0.0317 (14)	0.0391 (14)	0.0461 (16)	0.0020 (11)	0.0116 (12)	0.0033 (12)
C16	0.0303 (14)	0.0544 (17)	0.0416 (15)	−0.0049 (12)	0.0041 (12)	−0.0009 (13)
O2	0.0549 (12)	0.0499 (12)	0.0541 (12)	0.0140 (10)	0.0141 (10)	0.0128 (10)
C17	0.0378 (14)	0.0445 (14)	0.0333 (14)	−0.0052 (12)	0.0099 (12)	−0.0055 (12)
C5	0.0515 (17)	0.0402 (15)	0.0567 (18)	0.0131 (13)	0.0305 (15)	0.0087 (14)
C1	0.0313 (13)	0.0378 (14)	0.0345 (14)	0.0051 (11)	0.0097 (11)	0.0022 (11)
C11	0.0334 (13)	0.0392 (14)	0.0246 (12)	0.0019 (11)	0.0051 (10)	−0.0008 (10)
C10	0.0335 (14)	0.0436 (15)	0.0382 (14)	0.0122 (11)	0.0156 (11)	0.0069 (12)
C9	0.0411 (16)	0.0630 (18)	0.0353 (15)	0.0085 (14)	0.0109 (13)	0.0075 (14)
C12	0.0330 (13)	0.0333 (13)	0.0291 (13)	0.0007 (10)	0.0068 (11)	0.0016 (10)
C3	0.0440 (16)	0.0440 (16)	0.0580 (19)	−0.0035 (13)	0.0212 (14)	−0.0132 (14)
C14	0.0433 (16)	0.070 (2)	0.0392 (16)	0.0070 (15)	0.0105 (13)	−0.0146 (14)
C21	0.0397 (15)	0.0441 (15)	0.0454 (16)	0.0060 (12)	0.0157 (12)	0.0023 (13)
C18	0.0428 (16)	0.0471 (16)	0.0471 (16)	0.0020 (13)	0.0168 (13)	0.0111 (13)
C6	0.082 (2)	0.057 (2)	0.072 (2)	0.0237 (18)	0.049 (2)	0.0276 (18)
C13	0.0338 (15)	0.081 (2)	0.0364 (15)	0.0038 (14)	0.0007 (12)	−0.0137 (15)
C2	0.0356 (14)	0.0398 (14)	0.0391 (15)	0.0037 (11)	0.0107 (12)	−0.0026 (12)
C4	0.0603 (19)	0.0344 (15)	0.080 (2)	−0.0028 (14)	0.0423 (18)	−0.0024 (15)
C20	0.0407 (16)	0.0611 (19)	0.0580 (19)	0.0124 (14)	0.0100 (14)	0.0038 (16)
C19	0.0565 (18)	0.0564 (18)	0.0485 (17)	0.0101 (15)	0.0174 (14)	0.0177 (14)
C8	0.0511 (18)	0.095 (3)	0.0367 (16)	0.0188 (18)	0.0151 (14)	0.0153 (16)
C7	0.080 (2)	0.089 (3)	0.052 (2)	0.036 (2)	0.0372 (19)	0.037 (2)

### Geometric parameters (Å, °)

**Table:**

Br1—C15	1.904 (3)	C9—H9A	0.9300
O1—C2	1.362 (3)	C12—C13	1.386 (4)
O1—H1A	0.8200	C3—C4	1.354 (4)
N1—C18	1.477 (3)	C3—C2	1.402 (4)
N1—C21	1.477 (3)	C3—H3A	0.9300
N1—C11	1.487 (3)	C14—C13	1.378 (4)
C15—C16	1.369 (4)	C14—H14A	0.9300
C15—C14	1.373 (4)	C21—C20	1.506 (4)
C16—C17	1.379 (4)	C21—H21A	0.9700
C16—H16A	0.9300	C21—H21B	0.9700
O2—C20	1.418 (4)	C18—C19	1.512 (4)
O2—C19	1.420 (3)	C18—H18A	0.9700
C17—C12	1.379 (3)	C18—H18B	0.9700
C17—H17A	0.9300	C6—C7	1.359 (5)
C5—C6	1.414 (4)	C6—H6A	0.9300
C5—C4	1.417 (4)	C13—H13A	0.9300
C5—C10	1.421 (4)	C4—H4A	0.9300
C1—C2	1.382 (4)	C20—H20A	0.9700
C1—C10	1.443 (4)	C20—H20B	0.9700
C1—C11	1.530 (4)	C19—H19A	0.9700
C11—C12	1.523 (3)	C19—H19B	0.9700
C11—H11A	0.9800	C8—C7	1.394 (5)
C10—C9	1.414 (4)	C8—H8A	0.9300
C9—C8	1.372 (4)	C7—H7A	0.9300
			
C2—O1—H1A	109.5	N1—C21—H21A	109.8
C18—N1—C21	107.3 (2)	C20—C21—H21A	109.8
C18—N1—C11	113.12 (19)	N1—C21—H21B	109.8
C21—N1—C11	111.10 (19)	C20—C21—H21B	109.8
C16—C15—C14	121.1 (3)	H21A—C21—H21B	108.2
C16—C15—Br1	119.4 (2)	N1—C18—C19	109.5 (2)
C14—C15—Br1	119.4 (2)	N1—C18—H18A	109.8
C15—C16—C17	119.2 (2)	C19—C18—H18A	109.8
C15—C16—H16A	120.4	N1—C18—H18B	109.8
C17—C16—H16A	120.4	C19—C18—H18B	109.8
C20—O2—C19	110.1 (2)	H18A—C18—H18B	108.2
C16—C17—C12	121.3 (2)	C7—C6—C5	121.3 (3)
C16—C17—H17A	119.3	C7—C6—H6A	119.3
C12—C17—H17A	119.3	C5—C6—H6A	119.3
C6—C5—C4	121.5 (3)	C14—C13—C12	121.4 (3)
C6—C5—C10	119.4 (3)	C14—C13—H13A	119.3
C4—C5—C10	119.1 (3)	C12—C13—H13A	119.3
C2—C1—C10	118.5 (2)	O1—C2—C1	123.3 (2)
C2—C1—C11	121.2 (2)	O1—C2—C3	115.0 (2)
C10—C1—C11	120.2 (2)	C1—C2—C3	121.7 (3)
N1—C11—C12	111.41 (19)	C3—C4—C5	121.1 (3)
N1—C11—C1	110.96 (19)	C3—C4—H4A	119.4
C12—C11—C1	109.3 (2)	C5—C4—H4A	119.4
N1—C11—H11A	108.3	O2—C20—C21	112.0 (2)
C12—C11—H11A	108.3	O2—C20—H20A	109.2
C1—C11—H11A	108.3	C21—C20—H20A	109.2
C9—C10—C5	117.8 (2)	O2—C20—H20B	109.2
C9—C10—C1	123.0 (2)	C21—C20—H20B	109.2
C5—C10—C1	119.2 (2)	H20A—C20—H20B	107.9
C8—C9—C10	120.7 (3)	O2—C19—C18	112.3 (2)
C8—C9—H9A	119.6	O2—C19—H19A	109.1
C10—C9—H9A	119.6	C18—C19—H19A	109.1
C17—C12—C13	118.0 (2)	O2—C19—H19B	109.1
C17—C12—C11	120.4 (2)	C18—C19—H19B	109.1
C13—C12—C11	121.6 (2)	H19A—C19—H19B	107.9
C4—C3—C2	120.4 (3)	C9—C8—C7	121.3 (3)
C4—C3—H3A	119.8	C9—C8—H8A	119.3
C2—C3—H3A	119.8	C7—C8—H8A	119.3
C15—C14—C13	118.9 (3)	C6—C7—C8	119.4 (3)
C15—C14—H14A	120.5	C6—C7—H7A	120.3
C13—C14—H14A	120.5	C8—C7—H7A	120.3
N1—C21—C20	109.5 (2)		

### Hydrogen-bond geometry (Å, °)

**Table:**

*D*—H···*A*	*D*—H	H···*A*	*D*···*A*	*D*—H···*A*
O1—H1A···N1	0.82	1.93	2.622 (3)	142
C13—H13A···O1^i^	0.93	2.53	3.357 (4)	148

Symmetry codes: (i) −*x*+1, −*y*+1, −*z*.
